# Cellular Uptake of Silica and Gold Nanoparticles Induces Early Activation of Nuclear Receptor NR4A1

**DOI:** 10.3390/nano12040690

**Published:** 2022-02-18

**Authors:** Mauro Sousa de Almeida, Patricia Taladriz-Blanco, Barbara Drasler, Sandor Balog, Phattadon Yajan, Alke Petri-Fink, Barbara Rothen-Rutishauser

**Affiliations:** 1 Adolphe Merkle Institute, University of Fribourg, Chemin des Verdiers 4, 1700 Fribourg, Switzerland; mauro.sousadealmeida@unifr.ch (M.S.d.A.); patricia.taladriz@inl.int (P.T.-B.); barbara.drasler@unifr.ch (B.D.); sandor.balog@unifr.ch (S.B.); phattadon.yajan@unifr.ch (P.Y.); alke.fink@unifr.ch (A.P.-F.); 2 Water Quality Group, International Iberian Nanotechnology Laboratory (INL), Av. Mestre José Veiga s/n, 4715-330 Braga, Portugal; 3 Department of Chemistry, University of Fribourg, Chemin du Musée 9, 1700 Fribourg, Switzerland

**Keywords:** nanoparticles, gene regulation, endocytosis, inflammation, NR4A1

## Abstract

The approval of new nanomedicines requires a deeper understanding of the interaction between cells and nanoparticles (NPs). Silica (SiO_2_) and gold (Au) NPs have shown great potential in biomedical applications, such as the delivery of therapeutic agents, diagnostics, and biosensors. NP-cell interaction and internalization can trigger several cellular responses, including gene expression regulation. The identification of differentially expressed genes in response to NP uptake contributes to a better understanding of the cellular processes involved, including potential side effects. We investigated gene regulation in human macrophages and lung epithelial cells after acute exposure to spherical 60 nm SiO_2_ NPs. SiO_2_ NPs uptake did not considerably affect gene expression in epithelial cells, whereas five genes were up-regulated in macrophages. These genes are principally related to inflammation, chemotaxis, and cell adhesion. Nuclear receptor NR4A1, an important modulator of inflammation in macrophages, was found to be up-regulated. The expression of this gene was also increased upon 1 h of macrophage exposure to spherical 50 nm AuNPs and 200 nm spherical SiO_2_ NPs. NR4A1 can thus be an important immediate regulator of inflammation provoked by NP uptake in macrophages.

## 1. Introduction

Administration of clinically relevant nanoparticles (NPs) to humans can occur in various ways, including inhalation, oral ingestion, injection (intravenous, intramuscular, and subcutaneous), and dermal and ocular penetration [1,2]. Once inside the human body, the NPs can overcome organs and tissue barriers and then come into contact with single cells. Strong evidence has indicated that cellular responses to NPs are cell-type- and NP-dependent [1,3,4]. This means that each type of NP, with its intrinsic properties (e.g., size, shape, stiffness, surface chemistry, etc.), may lead to different cellular responses in different cell types [1,3,4,5]. For the design and optimization of biomedically relevant NPs, it is important to understand the mechanisms induced at the single-cell level. Cell-NP interaction may activate signaling cascades, leading to structural modifications inside cells and at the cell surface, interfering with normal cell function [6].

When NPs are deposited on the outer cellular membrane, they may interact and be internalized, mainly via endocytosis [7]. Endocytosis occurs via multiple mechanisms, including phagocytosis and pinocytosis (macropinocytosis, clathrin-mediated endocytosis (CME), caveolae-mediated endocytosis, and clathrin- and caveolae-independent endocytosis) [8]. All of the aforementioned mechanisms are complex and involve a wide range of molecules (e.g., surface receptors, lipids, and adaptor proteins) that work together to ensure an efficient process of endocytosis [9]. For example, non-porous silica (SiO_2_) and gold (Au) NPs, which have been extensively studied in biomedical context thanks to their controllable and large-scale syntheses, facile surface modification and biocompatibility, revealed different uptake mechanisms in different cell types [10,11]. Shapero et al. reported that spherical 50, 100, and 300 nm SiO_2_ NPs do not enter human lung epithelial cells(A549) via clathrin- or caveolae-mediated endocytosis. However, independent of their size, all NPs were internalized via an energy-dependent mechanism and ended up in lysosomes [12]. On the other hand, a similar study led by Hsiao et al. concluded that spherical 15, 60, and 200 nm SiO_2_ NPs are internalized via clathrin-mediated endocytosis in A549 cells, but also in macrophage-derived THP-1 cells [13]. The authors also proved that caveolae-mediated endocytosis contributes to the uptake of the 200 nm SiO_2_ NPs in A549 cells and the uptake of 60 and 200 nm NPs in macrophage-like THP-1 cells. In this study, the exposure of cells to NPs was conducted in the absence of serum, which might explain the different findings. Clearly, both studies prove that the presence of proteins in the cell-culture medium influence NP uptake and support the idea that observations in one type of cell should not be extrapolated to another. Spherical-shaped SiO_2_ NPs have received the most attention in nanomedicine; however it is known that NPs’ shape can also influence the cellular internalization mechanism [7]. Similarly, while spherical AuNPs are the main investigated type of NPs, several other studies looked into the effect of other shapes, such as rods, stars, and triangles, on the cellular uptake mechanisms. Ding et al. conducted a study to evaluate the effect of the cellular internalization of AuNPs in the form of spheres, rods, and stars in mouse breast cancer (4T1), human hepatoma (SMCC-7721), and human gastric mucosal (GES-1) cells [11]. The results showed that spherical AuNPs (average diameter of 15 and 45 nm), rod-shaped AuNPs (33 × 10 nm), and star-shaped AuNPs (average diameter 15 nm) were principally internalized through clathrin-mediated endocytosis in the different cell types. In addition, authors revealed that the uptake of Au nanostars also occurred via caveolin-mediated endocytosis, and that macropinocytosis was involved in the internalization of larger spherical AuNPs (average diameter 80 nm).

Aside from the internalization mechanism, several researchers have focused their investigations on different biological effects of SiO_2_ and Au NPs, such as cell viability, oxidative stress, and pro-inflammation [14,15,16,17]. Lin et al. reported an increase in intracellular reactive oxygen species (ROS), leading to oxidative stress and apoptosis after 12 h exposure of A549 cells to spherical 37 nm SiO_2_ NPs at 50 μg/mL [18]. Zhao et al. concluded that spherical SiO_2_ NPs of 27 nm can block the autophagic flux and impair lysosomal acidification in A549 cells after 24 h exposure to 50 μg/mL [19]. Kusaka et al. found that exposure of bone marrow-derived macrophages to 100 μg/mL of spherical SiO_2_ NPs with 30, 100, and 300 nm for 4 h, trigger inflammation, lysosomal destabilization, and cell death [20]. In summary, the recent in vitro experiments revealed that SiO_2_ NPs can impair normal cell function, inducing autophagic dysfunction, oxidative stress, and inflammatory response in different cell types in a dose and size-dependent manner. The exposure of different cells to AuNPs also revealed potential harmful effects. Uboldi et al. showed that 72 h exposure of spherical AuNPs with 9.5, 11.2, and 25 nm to A549 cells, at ~138 μg/mL, induced cytotoxicity [17]. Contrarily, Zhang et al. did not observe any sign of cytotoxicity and inflammation in murine macrophage cells (RAW 264.7) after 48 h exposure to spherical 60 nm AuNPs (100 μg/mL) [21]. Another study from D. Bachand et al. concluded that the exposure of spherical 20 and 60 nm AuNPs at concentrations of ~350 pg/mL did not cause any significant change in neither oxidative stress, nor cytotoxicity after 24 and 48 h in A549 cells [22]. Nevertheless, an increase in IL-8 secretion was observed for both AuNPs, revealing that these NPs can trigger inflammation. A higher IL-8 release from A549 cells was observed for 20 nm AuNPs, confirming that NP size affects the cellular responses. In short, cellular responses are influenced not only by the physicochemical properties of the NPs and the cell type, but also by other factors such as administered and delivered dose, and exposure time. Omics-based research has been applied in some of the aforementioned studies as it allows a thorough and systematic investigation of the changes that occur at the gene/transcript/protein level. This is crucial for a deeper understanding of the potential molecular mechanisms associated with NP uptake and potential adverse effects. Thus far, most of the research in this area has focused on the analysis of the transcriptome profiling after extended exposure to NPs (i.e., 24, 48, and 72 h). Nevertheless, we presume that cellular responses to NPs start a few minutes/hours after exposure, as cellular internalization occurs within this timeframe [12,23].

In this study, we evaluated the overall impact of spherical SiO_2_ NPs in human macrophages and lung epithelial cells, by using RNA-Seq to examine genome-wide transcriptional changes with a focus on endocytic and early-response genes. Based on previous findings [12,24] and on the in vitro sedimentation, diffusion, and dosimetry model (ISDD)[25], we showed that a small fraction of administered NPs can reach the cells after 1 h and be internalized. In this sense, we have decided to evaluate the cellular effects of SiO_2_ NPs exposure at three different time points: 1, 6, and 24 h. The current study is one of the first to investigate whether NP uptake influences the expression of endocytic genes at early time points. Our results show that the uptake of 60 nm SiO_2_ NPs did not affect the expression of endocytosis-related genes in macrophages and lung epithelial cells, but did increase the expression of five genes involved in inflammation, chemotaxis, and cell adhesion in macrophages. In particular, the most relevant gene involved in inflammation, nuclear receptor 4A1 (*NR4A1*), was up-regulated in macrophages upon 1 h exposure to 60 nm and 200 SiO_2_ NPs. Furthermore, to study the effect of material composition, macrophages were exposed for 1 h to spherical 50 nm Au NPs, which resulted in the up-regulation of *NR4A1*, revealing *NR4A1* as an early response gene and possibly an immediate regulator of inflammation.

## 2. Materials and Methods

### 2.1. Synthesis of Nonporous SiO_2_-Rhodamine B NPs

SiO_2_ NPs measuring 60 nm were synthesized following a modified-Stöber method [26,27]. Briefly, a mixture of absolute ethanol (144 mL, EtOH, ≥99.8%VWR, Dietikon, Switzerland), Milli-Q water (6.75 mL), and ammonium hydroxide (3.9 mL, NH_4_OH ≥25% NH_3_ in water, Merck, Zug, Switzerland was heated in a 500 mL rounded-bottom flask provided with a reflux system at 60°C under magnetic stirring. After 30 min, tetraethyl orthosilicate (11 mL, TEOS, >99%, Sigma Aldrich, Buchs, Switzerland) was added to the mixture and stirred for 2 min. Then, 100 µL of a mixture containing 100 µL Rhodamine B isothiocyanate (10 mg/mL RhoB in EtOH, Dye content ~95%, Sigma Aldrich, Buchs, Switzerland) and 1.5 µL of (3-aminopropyl) triethoxysilane (APTES, 99%, Sigma Aldrich, Buchs, Switzerland) were added to the flask using a syringe. After 4 h at 60 °C, the fluorescently labeled NPs were dialyzed against water for three days and stored at 4 °C in the darkness. The 200 nm SiO_2_ NPs were prepared using a similar synthetic approach. A mixture of 11 mL of TEOS, 180 mL of EtOH, 36 mL of Milli-Q water, and 24 mL of NH_4_OH was stirred at room temperature for 2 min before adding 300 µL of an APTES-RhodB mixture (10 mg/mL RhodB in EtOH) and 7.5 µL of APTES. The mixture was stirred overnight, and the particles were cleaned twice by centrifugation at 100 g and redispersed in EtOH. Three additional washes were carried out by centrifugation at 988 g to finally redisperse the NPs in autoclaved Milli-Q water. The concentration of the particles was determined by measuring the weighted average of dried 1 mL particle suspension in three different Eppendorf tubes.

### 2.2. Synthesis of Au NPs

Au NPs measuring 50 nm were synthesized by the Brown method [28,29]. Briefly, 1.34 mL of 0.22 M of hydroxylamine hydrochloride (NH_2_OH ∙HCl, ACS reagent, 98%, Sigma-Aldrich, Buchs, Switzerland) was added to a solution containing 144 mL of gold (III) chloride trihydrate (0.25 mM, HAuCl_4_∙3H_2_O, ≥99.9%, Sigma-Aldrich, Buchs, Switzerland), as-prepared 15 nm gold seeds ([Au] = 0.0125 mM) and sodium citrate tribasic dihydrate (0.5 mM NaCit, C_6_H_5_Na_3_O_7_∙ 2H_2_O, ≥98%, Sigma-Aldrich, Buchs, Switzerland) under vigorous magnetic stirring. After 15 min under magnetic stirring, the NPs were cleaned by centrifugation at 3500 rpm for 20 min and redispersed in 0.5 mM NaCit. Au seeds measuring 15 nm were prepared by the well-known Turkevich method [30]. Briefly, 0.5 mM HAuCl_4_ was boiled in the presence of 1.7 mM NaCit for 15 min. Au NPs measuring 15 nm were cooled down to room temperature and stored at 4°C overnight before using them to synthesize the larger particles.

### 2.3. Fluoresbrite^®^ Yellow-Green Polystyrene (PS)-Based Latex NPs

Yellow-green PS microspheres with ~50 nm in diameter were purchased from Chemie Brunschwig AG (Basel, Switzerland). The company states that PS NPs are stable and dye leaching is not expected, making them suitable for use in cell experiments.

### 2.4. Physicochemical Characterization

NPs were drop-cast onto a 300-mesh carbon-membrane-coated copper grid and imaged using a Tecnai Spirit transmission electron microscope (TEM) (FEI, Hillsboro, OR, USA) operating at 120 kV equipped with a CCD camera (Eagle, Thermo Fischer, Waltham, MA, USA). The core diameter and size distribution were calculated using an open-source image processing program (ImageJ). UV−Vis extinction spectrum of Au NPs was recorded in a Jasco V-670 spectrophotometer using 10 mm path length quartz Suprasil-grade cuvettes (Hellma Analytics, Plainview, NY, USA) at 25 °C. The stability of the NPs in the cell culture media was tested at 0 and 24 h by DLS at 25 °C and one scattering angle (90°) using a commercial goniometer instrument (3D LS Spectrometer, LS Instruments AG, Fribourg, Switzerland) equipped with a linearly polarized and a collimated laser beam (Cobolt 05-01 diode-pumped solid-state laser, λ = 660 nm, P max. = 500 mW). Two APD detectors, assembled for pseudo-cross-correlation, were used to improve the signal-to-noise ratio. The scattering signal of complete RPMI 1640 (cRPMI 1640) and serum-free RPMI 1640 (i.e., without Fetal Bovine Serum (FBS) but supplemented with L-glutamine and penicillin/streptomycin) obtained by DLS was subtracted from the signal of the NPs suspended in the media, as presented elsewhere [31], to obtain only the contribution of the particles. NPs dispersed in Milli-Q water were used as a control. The mean and standard deviation were calculated from five independent measurements. The surface charge was determined by phase-amplitude light scattering (ZetaPALS, Brookhaven Instruments Corp., Holtsville, NY, USA) in Milli-Q water.

### 2.5. Cell Culture

Cell culture reagents were purchased from Gibco, Thermo Fisher Scientific (Zug, Switzerland), unless otherwise specified. Human alveolar epithelial type II cells (A549 cell line) from American Type Culture Collection (ATCC, Rockville, MD, USA) were cultured in Roswell Park Memorial Institute (RPMI)-1640 cell culture medium supplemented with 10 vol.% FBS, 2 mM L-Glutamine (100 Units/mL), and Penicillin-Streptomycin (100 µg/mL). The final solution is referred to as cRPMI 1640. Cell cultures were kept in a humidified incubator (37°C, 5% CO_2,_ and 95% humidity). A549 epithelial cells were sub-cultured twice per week using a mixture of 0.25% Trypsin-Ethylenediaminetetraacetic acid (EDTA) according to the ATCC recommendations. Prior to seeding, cell concentration was determined using the trypan blue exclusion assay (0.4%vol. in phosphate-buffered saline solution (PBS, pH 7.2, Gibco, Life Technologies Europe B.V., Zug, Switzerland)) and an automated cell counter (EVE, NanoEnTek Inc., Seoul, South Korea). A549 cells (5.26 × 10^4^ cells/cm^2^), in the passage range of 4–15, were seeded for 24 h in cRPMI 1640 followed by serum starvation for 24 h before NP exposure. Serum starvation was performed to synchronize all cells to the same cell cycle phase. Primary human monocyte-derived macrophages (MDMs) were obtained by isolating and further differentiating human peripheral blood monocytes from human blood buffy coats (Blood Donation Service, Bern University Hospital, Bern, Switzerland), as previously described [32,33]. The work involving primary monocytes isolation from human blood was approved by the committee of the Federal Office for Public Health Switzerland (reference number: 611-1, Meldung A110635/2). Briefly, human blood was separated using density gradient filtration (Lymphoprep, Grogg Chemie, Stettlen, Switzerland) and the monocyte fraction was extracted from the mixture and purified using CD14+ magnetic MicroBeads (Miltenyi Biotec GmbH, Bergisch Gladbach, Germany) according to the manufacturer’s protocol. For the monocyte differentiation, the isolated blood monocytes were cultured in 6-well plates (Corning^®^Falcon, Reinach, Switzerland) containing 3 mL of cRPMI 1640 supplemented with the macrophage colony-stimulating factor (M-CSF) (10 ng/mL, Milteny Biotech, Bergisch Gladbach, Germany) for seven days at a density of 10^6^ cells/mL. After this period, cRPMI containing the M-CSF was removed and MDMs (1.05 × 10^5^ cells/cm^2^) were seeded for 24 h before NP exposure.

### 2.6. NPs Exposure

A549 and MDMs grown in 6-well plates and 35 mm glass-bottom dish (MatTek Inc, Ashland, MA, US) were exposed to 3 mL of 60 nm SiO_2_-RhodB, 200 nm SiO_2_-RhodB, 60 nm PS particles ([NP] = 20 µg/mL), or Au NPs ([Au] = 20 µg/mL) previously suspended in cRPMI 1640. For experiments where µ-Slide 8 Wells (Ibidi, Graefelfing, Germany) were used (i.e., sections “Fluorescence imaging” and “Co-localization analysis”), cells were exposed to 316 µL of previously suspended NPs. ISDD model was used to estimate the particle deposition [25]. The relative densities and the diameter of each NP, based on TEM analysis, were taken in consideration. Amorphous silica, 2.2 g/cm^3^; gold, 19.32 g/cm^3^; polystyrene, 1.05 g/cm^3^. After exposure, cells were washed 3 times with PBS to remove the non-cell adhered NPs.

### 2.7. Cytotoxicity Assay

Lactate dehydrogenase (LDH, Roche Applied Science, Mannheim, Germany) assay was performed on the cell supernatants after NPs exposure in 6-well plates. Triton X-100 at 0.2 vol.% was added to the cell culture medium as a positive control for 6 h prior to collecting the supernatant. LDH levels were measured in triplicate by following the manufacturer’s protocol. The absorbance of the colorimetric product was determined by spectrophotometry (Benchmark Microplate reader, BioRad, Cressier, Switzerland) at 490 nm with a reference wavelength of 630 nm. Interference analysis was performed for SiO_2_ NPs as recommended by Petersen et al. [34], at the administered dose (20 µg/mL), in one independent experiment with 3 technical replicates. There was no evidence of quenching or auto-absorption.

### 2.8. Flow Cytometry

After cell growth and NPs exposure for 1, 6, and 24 h in 6-well plates, MDMs were scraped off in 1 mL of cRPMI, using a cell scraper (Sarstedt, Sevelen, Switzerland) and collected in a flow cytometry tube (5 mL Polystyrene Round-Bottom Tube, Corning^®^ Falcon, Reinach, Switzerland). A549 cells were detached with Trypsin-EDTA (300 µL) for 6 min followed by the addition of 700 µL of cRPMI 1640. Cells were centrifuged at 4 °C for 5 min at 300 *g*, washed 2 times in PBS and then resuspended and fixed with 2 vol.% paraformaldehyde (PFA, Sigma-Aldrich, Buchs, Switzerland) in PBS for 15 min at 4 °C. Two additional washing steps were performed in PBS, before resuspension in cold FC buffer (PBS with 1 w/v.% bovine serum albumin (BSA, Sigma-Aldrich, Buchs, Switzerland), 0.1 vol.% sodium azide (Sigma-Aldrich, Buchs, Switzerland), and 1 mM EDTA (Sigma-Aldrich, Buchs, Switzerland) at pH 7.4. Data acquisition was performed on a BD LSR FORTESSA (BD Biosciences, San Jose, CA, USA) equipped with a yellow-green laser and PE filter where 30,000 events were recorded. Flow cytometry data was analyzed using the FlowJo software v.10.

### 2.9. Fluorescence Imaging

After NPs exposure for 1, 6, and 24 h, cells were fixed with 4 vol.% PFA in PBS for 15 min at room temperature and permeabilized for 10 min in 0.2 vol.% Triton X-100 in PBS. Samples were washed thrice with PBS between steps. F-actin was stained with Alexa Fluor 488 Phalloidin (0.66 µM in PBS, Cat. # A12379, Invitrogen, Thermo Fisher Scientific Inc., Zug, Switzerland) for 1 h, and cell nuclei counterstained using 4′,6-diamidino-2-phenylindole (DAPI, 1 μg/mL in PBS, Sigma-Aldrich, Buchs, Switzerland) for 5 min in PBS. Samples were washed 3 times using PBS and kept in PBS until further analysis. During fixation and staining, samples were kept at room temperature and dark conditions between steps. Images were acquired in an inverted Zeiss LSM 710 Meta apparatus (Axio Observer.Z1, Zeiss, Oberkochen, Germany) using an excitation laser of 405 nm (DAPI), 488 nm (Alexa Fluor 488), and 561 nm (rhodamine B) equipped with a Plan-Apochromat 63x/1.4 Oil M27 objective (Zeiss GmbH, Oberkochen, Germany).

### 2.10. Co-Localization Analysis

Co-localization of the exposed NPs with early endosomes was evaluated after 1 and 6 h. NPs co-localization with lysosomes was studied at 6 and 24 h. After NPs exposure, cells were washed 3 times with PBS and incubated with fresh cRPMI supplemented with 75 nM LysoTracker Green DND-26 (Invitrogen, Thermo Fisher Scientific Inc., Zug, Switzerland) for 15 min to stain the lysosomes. Then, the cells were washed twice with PBS and immediately imaged after the addition of cRPMI. For early endosomes labeling, immunostaining with early endosome antigen 1 (EEA1) was performed. Cells were fixed and permeabilized, as mentioned in the previous section. After, 20 µg/mL of EEA1 (Cat. # ab109110, Abcam, Cambridge, UK) in antibody solution (1 w/v.% bovine serum albumin (BSA, Cat. # A7030, Sigma-Aldrich, Zug, Switzerland) and 0.1 vol.% Triton-X in PBS) was added for 2 h. A secondary antibody goat anti-rabbit Alexa Fluor 647 (2 µg/mL, Cat. # A21244, Invitrogen, Thermo Fisher Scientific Inc., Zug, Switzerland) in antibody solution was added for 1 h. Finally, cells were counterstained with DAPI. All steps were conducted at room temperature and under dark conditions. The NPs co-localization was calculated using the Pearson’s correlation coefficient (PCC) and the open source plugin for ImageJ, EzColocalization [35].

### 2.11. Dye Leaching from SiO_2_-Rho B NPs

The potential release of dye from SiO_2_ NPs in cell culture medium and lysosomal milieu was investigated by incubating the NPs in cRPMI (without phenol red) and artificial lysosomal fluid (ALF). ALF was prepared as previously reported [36]. Briefly, sodium chloride (3.210 g), sodium hydroxide (6.000 g), citric acid (20.800 g), calcium chloride (0.097 g), sodium phosphate heptahydrate (0.179 g), sodium sulfate (0.039 g), magnesium chloride hexahydrate (0.106 g), glycerin (0.059 g), sodium citrate dihydrate (0.077 g),sodium tartrate dihydrate (0.090 g), sodium lactate (0.085 g), sodium pyruvate (0.086 g), and formaldehyde (1.000 mL, added fresh before use) were dissolved in 200 mL of MilliQ water to obtain a 5× stock solution. The stock solution was later diluted with MilliQ water and NPs to a final concentration of 20 µg/mL. NPs were incubated in cRPMI and ALF for 24 h at 37 °C. Then, the NPs were centrifuged at high speed (16,000 *g*) for 1 h and the supernatants were collected. The supernatants were centrifuged again at the same speed, to guarantee that a minimum number of particles remains in suspension. A control containing the NPs in water, at the administered dose (20 µg/mL), was included in the experiments. Fluorescence emission intensity was measured on a Fluorolog TCSPC spectrofluorometer (Horiba, Northampton, UK) with the FluorEssence software (v3.8). For each sample, emission spectrum with a λem between 560 to 700 nm and a fixed λex of 550 nm, was recorded. The excitation and emission slits were fixed to 4 nm.

### 2.12. Focused Ion Beam-Scanning Electron Microscopy (FIB-SEM)

Cells were seeded in a 35 mm glass-bottom dish (MatTek Inc, Ashland, MA, USA), exposed to SIO_2_ NPs for 6 h and fixed with 2 vol.% PFA and 2.5 vol.% glutaraldehyde (25%, Electron Microscopy Sciences, Hatfield, PA, USA) in PBS for 3 h on ice. Samples were then treated with a mixture of 3 w/v.% potassium hexacyanoferrate(II) trihydrate (≥99.95%, Sigma Aldrich, Buchs, Switzerland) and 2 vol.% osmium tetroxide (4% in H_2_O, Sigma Aldrich, Buchs, Switzerland) in Milli-Q water, together with 0.2 M cacodylate buffer (Polysciences, Eppelheim, Germany) for 1 h at room temperature. After, a treatment with 1 w/v% thiocarbohydrazide (98%, Sigma Aldrich, Buchs, Switzerland) in Milli-Q water was performed for 20 min at 60 °C. Finally, samples were incubated with 2 vol.% osmium tetroxide in Milli-Q water for 30 min. Samples were dehydrated using increasing graded ethanol series, followed by embedding and polymerization in Epon resin (Epoxy embedding kit, Sigma Aldrich, Buchs, Switzerland) for 48 h at 60 °C. Polymerized Epon blocks were then attached to an aluminum stub with carbon tape, and a thin layer of ~2 nm Au was sputtered onto the sample surface to render them conductive. FIB-SEM milling and imaging was performed using a Thermo Scientific Scios 2 Dual Beam microscope (Thermo Fisher Scientific, Waltham, MA, USA). A FIB operated at 30 kV was used to localize the cells of interest underneath the resin block. Once the region of interest was chosen, a trench was created using an ion (Ga^+^) beam (30 kV and current of 1 nA). A final polishing step was performed 1 nA at 30 kV. The freshly milled cross-section was imaged using an electron beam with an acceleration voltage of 2 kV and a current of 50 pA and the backscattered electron detector. This investigation was complemented by energy-dispersive x-ray spectroscopy (Thermo Fisher Scientific, Waltham, MA, USA) for the chemical analysis of silicon (Si).

### 2.13. Total RNA Isolation and Illumina Sequencing (RNA-Seq)

After NPs exposure, total RNAs were isolated from cells growing in 6-well plates. Cell lysis was performed directly in the well, using 250 μL of BL + TG buffer (Promega Madison, WI, USA), and total RNA was extracted using ReliaPrep™ RNA Cell Miniprep System (Promega, Z6012, Madison, WI, USA) following the manufacturer’s protocol. The quantity and quality of RNA were examined by Thermo Scientific^TM^ NanoDrop^TM^ 2000 Spectrophotometer and Agilent 2100 Bioanalyzer (Agilent Technologies, Santa Clara, CA, USA). Only RNA with OD 260/280 ≥ 1.8 and RNA integrity number ≥ 7 were selected for the subsequent experiments. Equal quantities of high-quality RNA, i.e., that met the above-stated criteria, from each sample were pooled together for mRNA library preparation (TruSeq Stranded RNA) and sequencing (HiSeq400 SR 150) at the Genomic Technologies Facility, Lausanne, Switzerland. Statistical analysis was performed in R (R version 4.0.2). Genes with low counts were filtered out according to the rule of one count per million (1 cpm). Library sizes were scaled using TMM normalization. The normalized counts were transformed to cpm values, and a log2 transformation was applied by means of the function cpm with the parameter setting prior.count = 1 (EdgeR v 3.30.3) [37]. After data normalization, a quality control analysis was performed through hierarchical clustering and sample PCA. Differential expression was computed with the R Bioconductor package limma [38] by fitting data to a linear model. The approach limma-trend was used. Sample pairings were taken into account by including a factor in the model. Fold changes were computed, and a moderated *t*-test was applied. P-values were adjusted using the Benjamini-Hochberg (BH) method.

### 2.14. Real-Time qRT-PCR

The reverse transcriptase reaction was performed with the Omniscript RT system (Qiagen, Hilden, Germany), OligodT (Microsynth, Balgach, Switzerland), and RNasin Plus RNase Inhibitor (Promega, Madison, WI, USA, Switzerland). The synthesis of complementary DNA (cDNA) was performed by using 6.5 µL of isolated RNA (250 ng), 1 μL oligo-dT primer (10 μM), 0.25 µL RNase inhibitor, 1 µL dNTP Mix (5 mM), 0.25 µL Omniscript reverse transcriptase (1 Unit), and 1 µL buffer RT. The real-time PCR was performed on the 7500 fast real-time PCR system (Applied Biosystems, Thermo Fisher Scientific, Waltham, MA, USA) by mixing 2 μL 5-fold diluted cDNA with 5 μL SYBR-green master mix (Fast SYBR Green master mix, Applied Biosystems, Thermo Fisher Scientific, Waltham, MA, USA), 2 μL nuclease-free water (Promega, Madison, WI, USA), and 2 μL primer mix (91 nM). Relative expression levels were calculated using the Pfall method [39] with glyceraldehyde-3-phosphate dehydrogenase (GAPDH) and tyrosine 3-monooxygenase/tryptophan 5-monooxygenase activation protein zeta (YWHAZ) as internal standard genes. Primers were purchased from Thermo Fisher Scientific (Zug, Switzerland). Details about the primers are included in the (Supplementary Information Table S1).

### 2.15. Western Blot

Total protein was isolated from cells growing in 6-well plates. Cell lysis was performed directly in the wells by adding 50 µL of ice-cold T-PER buffer (Cat. # 78510, Thermo Fisher Scientific, Zug, Switzerland) supplemented with Halt^TM^ Protease Inhibitor Cocktail, EDTA-free (Cat. # 78425, Thermo Fisher Scientific, Zug, Switzerland) and sodium fluoride (Cat. # 27860, 20mM, VWR, Dietikon, Switzerland). Plates were kept at 4°C for 20 min. Protein lysates were pipetted up and down, transferred to 1.5 mL Eppendorf tubes, kept on ice for 10 min and centrifuged at 10,000 *g* for 5 min. The protein in the supernatant was collected and quantified via Bradford assay. The samples were boiled in a reducing Laemmli buffer for 5 min, and the same amount of protein was loaded in a 7.5% SDS-PAGE (Bio-Rad, Hercules, CA, USA). Proteins were electrophoretically transferred onto polyvinylidene difluoride (PVDF) membranes at 150 mA for 75 min under wet conditions. A solution of 0.1 w/v.% Ponceau S (Cat. # 141194, Sigma-Aldrich, Buchs, Switzerland) was used to confirm the transfer of proteins. A solution containing 3 w/v.% BSA and 0.1 vol.%Tween 20 (Cat. # P9416, Sigma-Aldrich, Buchs, Switzerland) in Tris-buffered saline (TBS) solution was used to block the nonspecific sites for 1 h. The same solution was used for immunostaining with primary and secondary antibodies. Three rounds of washing with TTBS (0.1 vol.% Tween 20 in TBS) were performed between steps. Primary antibody was added to the blots overnight at 4 °C. The following concentrations of antibodies were used: α-tubulin (1 µg/mL, Cat. # sc-32293, Santa Cruz Biotechnology, Heidelberg, Germany) GAPDH (1 µg/mL, sc-47724, Santa Cruz Biotechnology, Heidelberg, Germany), EEA1 (2 µg/mL, Cat. # ab109110, Abcam, Cambridge, UK), and Nur77 (2 µg/mL, Cat. # sc-365113, Santa Cruz Technology, Heidelberg, Germany). The blots were then incubated with a goat anti-mouse HRP conjugated secondary antibody (Cat. # HAF007, R&D, Abingdon, UK) for 1 h at 1:2000 (α-tubulin), 1:4000 (GAPDH), 1:1000 (EEA1), and 1:1000 (Nur77). A molecular weight marker mPAGE^®^ Color Protein Standard (Cat. # MPSTD4, Sigma-Aldrich, Buchs, Switzerland) was used to identify the corresponding detected bands. Protein bands were visualized using the chemiluminescent HRP detection reagent Immobilon Forte Western HRP substrate (Cat. # WBLUF0020, Sigma-Aldrich, Buchs, Switzerland). The optical density of the bands was estimated using ImageJ. The housekeeping proteins α-tubulin and GAPDH were used as normalization controls.

### 2.16. Statistical Analyses

Comparisons between two related groups were made by paired *t*-test. Two-way ANOVA (Dunnett’s post-hoc test for multiple comparisons) was used to compare more than two groups with more than one variable. Statistical analyses were performed with GraphPad Prism 9.2 software (GraphPad Software, San Diego, CA, USA).

## 3. Results and Discussion

### 3.1. Interaction and Localization of SiO_2_ NPs in MDMs and A549 Cells

**Figure 1:**
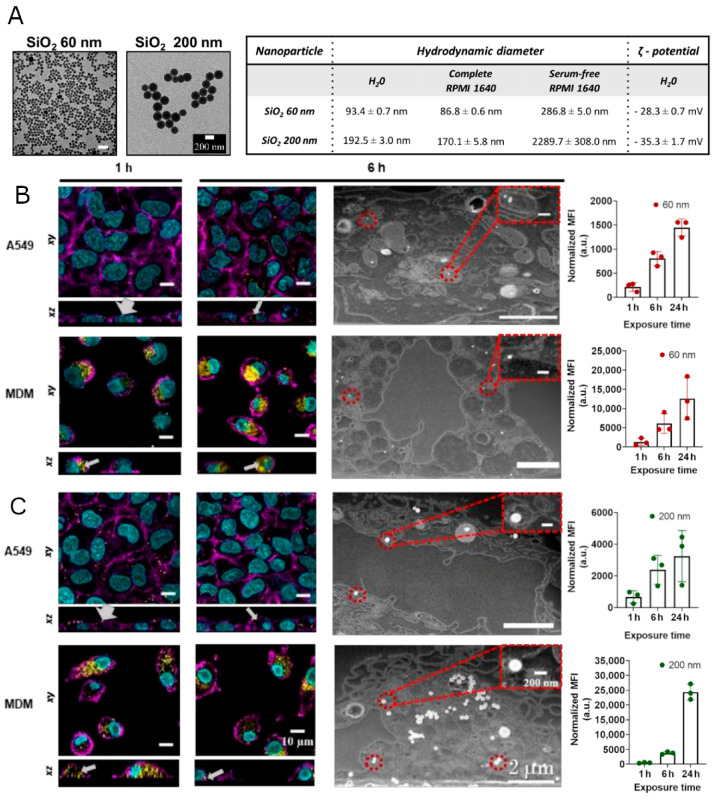
SiO_2_ NP characterization and cellular uptake. (**A**) Representative transmission electron microscopy (TEM) micrographs and physicochemical characterization of SiO_2_ NPs used in this study. Scale bar = 200 nm. Hydrodynamic diameter measured by dynamic light scattering in H_2_O, complete RPMI 1640, and serum-free RPMI 1640, revealing the aggregation of both NPs in serum-free RPMI 1640. Confocal laser scanning microscopy (CLSM), focused ion beam-scanning electron microscopy (FIB-SEM) micrographs and flow cytometry data (bar graphs on the left), revealing the association of 60 (**B**) and 200 nm (**C**) SiO_2_ with lung epithelial cells (A549) and primary human monocyte-derived macrophages (MDMs) after 1, 6, and 24 h of exposure to 20 µg/mL. Bar graphs represent the median fluorescence intensity (MFI), normalized to untreated cells. Data is presented as mean ± standard deviation (n = 3). Cell nuclei (cyan), cytoskeleton (magenta), and NPs (yellow). Thicker and thinner grey arrows indicate extracellular (surface bound) and intracellular localization of NPs, respectively. The red dashed circles indicate the intracellular localization of NPs. Scale bar = 10 µm for CLSM pictures. Scale bar = 2 µm for FIB-SEM pictures and scale bar = 200 nm for zoomed-in images.

Non-porous SiO_2_ NPs, measuring 60 and 200 nm in diameter, were synthesized and functionalized with the fluorescent dye RhoB, to enable their detection within cells using flow cytometry and confocal laser scanning microscopy (CLSM). Representative TEM micrographs of the individual particles and physicochemical properties are represented in Figure 1A and Figure S1. It has been demonstrated that cellular responses differ significantly after interacting with NPs in an aggregated form or as individual particles [40]. Therefore, the stability of both 60 and 200 nm SiO_2_ NPs in serum-free RPMI 1640 and complete RPMI 1640 (cRPMI) was evaluated by dynamic light scattering (DLS) at 24 h. An increase in size, a consequence of the aggregation of the NPs, was observed for both NPs in serum-free RPMI 1640, whereas in cRPMI the particles remain stable (Figure 1A inset table). The latter is explained by the ability of proteins in the serum to adsorb on the NPs surface, creating the so-called protein corona [41] and assisting NP stabilization via steric and/or hydration interactions [3]. Due to the aggregation of both SiO_2_ NPs in serum-free RPMI 1640, as demonstrated by DLS and CLSM (Figure S2), the exposure of cells to NPs was performed in cRPMI. In addition, the stability of the fluorescent probes on the SiO_2_ NPs in cRPMI and lysosomal fluid was evaluated (Figure S3). The performed fluorescence measurements revealed that there is a minor signal from the supernatants after SiO_2_ NPs incubation in cRPMI and ALF for 24 h at 37 °C. The findings confirm the stability of the fluorophore RhoB on SiO_2_ NPs.

MDM and A549 cells were selected for this study as MDMs are phagocytic cells and one of the first cell types to interact with NPs in the body, contributing to rapid NP clearance from the tissue where these cells reside [42]. The non-phagocytic A549 cell line is frequently used to mimic an alveolar type II epithelial barrier [43] and is one the most widely used cell lines in human research in a wide range of applications, including in the testing of novel drugs [44] and in particle uptake mechanism studies [14,45,46]. Initially, cellular cytotoxicity was evaluated using a lactate dehydrogenase assay after 6 and 24 h exposure (Figure S4). No significant alterations in cell membrane permeability after exposure to 60 nm and 200 nm SiO_2_ NPs were observed for either cell type, confirming their non-cytotoxicity.

The interaction and association (i.e., both plasma membrane-bound and internalized) of 60 and 200 nm SiO_2_ NPs with A549 and MDM cells were evaluated after 1, 6, and 24 h of NP exposure using CLSM, focused ion beam-scanning electron microscopy (FIB-SEM) and flow cytometry (Figure 1, Figures S5 and S6). CLSM and flow cytometry showed that the interaction between both cell types and NPs occurs within the first hour. However, in CLSM images, it is possible to conclude that the majority of NPs interact with the A549 cell membrane in the first hour but are not internalized. CLSM and FIB-SEM micrographs reveal the internalization of both SiO_2_ NPs by A549 cells after 6 h of exposure. In MDM cells, the association of SiO_2_ NPs begins within the first hour and increases over time. A high number of 60 and 200 nm SiO_2_ NPs can be seen inside MDMs at the 6 h timepoint. The association rate of NPs with MDM cells was higher than with A549 cells, which is to be expected based on the higher clearance capability of the latter cell type. In addition, cell division in dividing cells (A549) takes place and the NP load might thus be lower than in non-dividing cells (MDM) [47]. Macrophages are a type of cell of the reticuloendothelial system (RES) [1]. RES is the biggest limitation in NP drug delivery because it is one of the main factors responsible for the sequestration and clearance of NPs [48].

**Figure 2:**
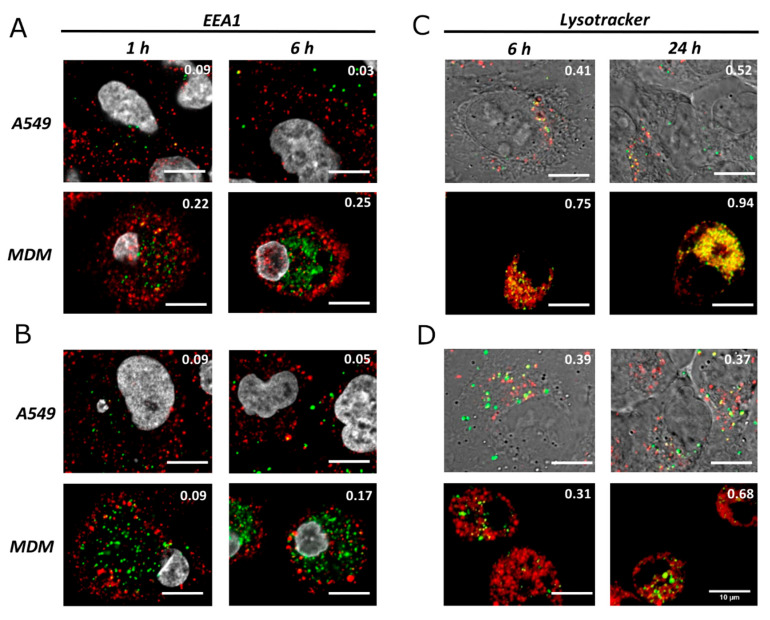
Co-localization of SiO_2_ NPs with early endosomes (EEA1, **A**,**B**) and lysosomes (Lysotracker, **C**,**D**) at different time points. SiO_2_ of 60 nm are represented in (**A**,**C**), and 200 nm in (**B**,**D**). Cell nuclei (grey), EEA1 and lysotracker (red), and NPs (green). The co-localization was determined by the Pearson correlation coefficient and values are represented at the top right of each image. Phase contrast images for NP-lysosome co-localization, in lung epithelial cells (A549), were included to facilitate cell structure visualization. Scale bar = 10 µm.

After uptake, it is expected that NPs will be localized within endocytic vesicles that fuse together with the early endosomes/phagosomes and, later, with the lysosomes [1]. With this in mind, the co-localization of SiO_2_ NPs with early endosomes and lysosomes was investigated. Our results (Figure 2) show a very weak co-localization (Pearson correlation coefficient (PCC) < 0.3) with early endosome antigen 1 (EEA1) for both 60 and 200 nm NPs in MDMs or A549 cells, confirming that SiO_2_ NPs only stay in the early endosomes for a short period of time. As described in the literature, early endosomes rapidly fuse with late endosomes, over an 8–15 min period [45], which is consistent with the obtained results. In contrast, higher PCCs were obtained for co-localization between lysosomes (Lysotracker) and SiO_2_ NPs. Higher co-localization values were obtained for 60 nm NPs in both MDMs and A549 cells, which can be rationalized by a different phagosomal/lysosomal transport mechanism of the NPs. The endosomes/phagosomes formed during NP uptake have varying sizes, which can strongly affect endosomal/phagosomal transport [46] and, consequently, maturation and fusion with lysosomes. After 24 h, there was an increase in co-localization of both particles with lysosomes in MDM cells, possibly due to accumulation of these particles in this compartment. Furthermore, the PCC is higher for both particle types at all investigated time points in MDMs, which can be related to the higher number of intracellular NPs.

### 3.2. Regulation of Gene and Protein Expression upon Uptake of SiO_2_ NPs

**Figure 3:**
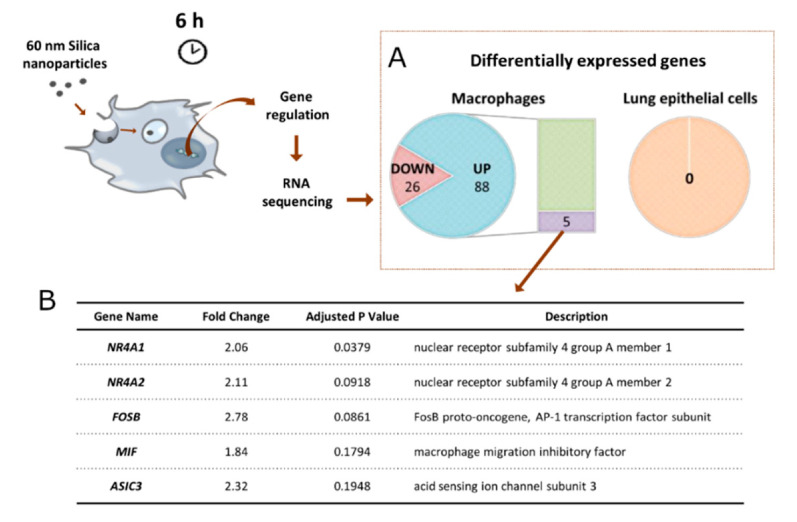
(**A**) Whole transcriptome screening via RNA sequencing upon exposure of A549 lung epithelial cells and primary human monocyte-derived macrophages (MDMs) to 60 nm SiO_2_ NPs for 6 h. Number of differentially expressed genes in lung epithelial cells and macrophages with an adjusted p-value of less than 0.2. No changes in the gene expression were observed for A549 cells. In MDM cells, 117 genes were found to be differentially regulated (26 down-regulated and 88 up-regulated), but only five revealed a greater than 1.5 fold change. (**B**) List with the five up-regulated genes in MDM cells.

To investigate the early changes that occur at the genetic level upon cell-NP uptake, A549 and MDMs were exposed to 60 nm SiO_2_ NPs for 6 h, followed by a genome-wide transcriptome analysis via RNA-seq (Figure 3). Only 60 nm SiO_2_ were included in this first screening. The results revealed that no gene was differentially expressed in A549 cells. On the other hand, 117 genes (adjusted p-value < 0.2) were found to be differentially expressed in MDM cells, but only five (*NR4A1, NR4A2, FOSB, MIF, and ASIC3*) showed a change greater than 1.5-fold. Surprisingly, none of these five genes are related to endocytic mechanisms. The most significantly changed gene, *NR4A1*, revealed a 2.06-fold change. *NR4A1* is an orphan nuclear receptor and is part of the nuclear receptor group 4A (NR4A) subfamily of nuclear hormone receptors [49]. It modulates the inflammatory response of macrophages through a number of mechanisms, including transcriptional reprogramming of mitochondrial metabolism [50]. The up-regulation of this gene can be triggered via physical stimulation and by inflammatory and growth factors [51]. Waters et al. also revealed the up-regulation of *NR4A1* in macrophages after 2 h of exposure to amorphous SiO_2_ NPs [52]. A different analytical technique, gene set enrichment analysis (GSEA), was performed for MDM cells, including all the differentially expressed genes, and the principal gene ontology (GO) biological processes (BPs) are shown in Figure S7. The uptake of 60 nm SiO_2_ NPs led to significant changes in the group of genes involved in cell–cell adhesion, cell chemotaxis, immune response, and inflammation. The changes in these processes have also been observed in previous studies [53,54,55]. These findings reveal that NP internalization does not lead to major transcriptional changes at early time points in the genes related to endocytosis, and lets us suggest that regulation might occur at the protein level (i.e., post-translation modifications). Furthermore, it demonstrates that NPs can cause inflammation even when they are not cytotoxic. The main reason for NP uptake in macrophages is their capability to recognize the opsonins present at NPs surface. The process of opsonization occurs upon NPs interaction with physiological fluids, containing different biomolecules including opsonins that promote cellular recognition and clearance by macrophages [7]. The opsonins at NPs surface can also dictate the extent of uptake and toxicity [42]. Fedeli et al. produced 26 nm spherical SiO_2_ NPs and proved that high amounts of histidine rich glycoprotein adsorbed at NPs surface avoided human macrophage recognition [56].

**Figure 4:**
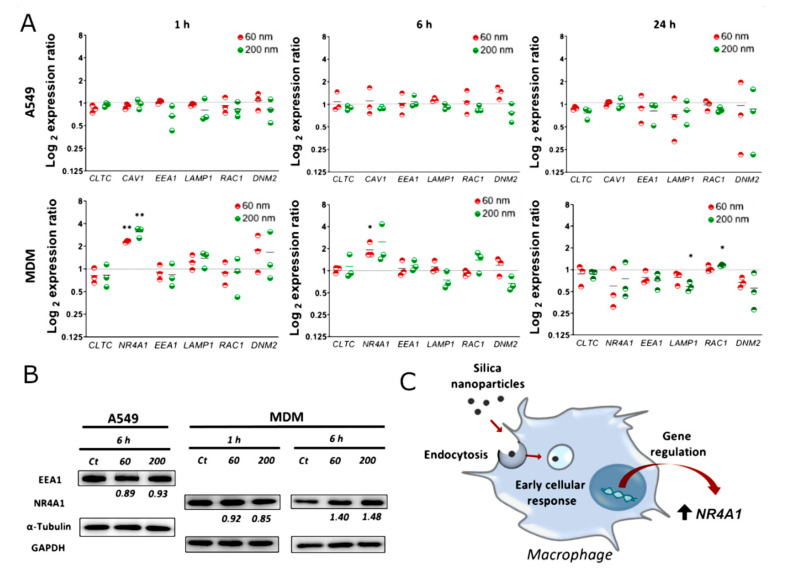
Gene and protein expression upon exposure to SiO_2_ NPs. (**A**) Real-time qRT-PCR results representing the expression of several genes upon exposure to 60 and 200 nm SiO_2_ NPs at different time points (1, 6, and 24 h) in lung epithelial cells (A549) and primary human monocyte-derived macrophages (MDMs). Statistically significant differences among the groups (Two-way ANOVA Dunnett’s post-hoc test for multiple comparisons): * p ≤ 0.05; ** p ≤ 0.01. (**B**) Expression of proteins NR4A1 and EEA1 were analyzed by Western blot. The representative images are shown. The mean expression ratios of the indicated protein, determined via densitometry from three independent experiments, are shown at the bottom of each blot. (**C**) Scheme representing the early activation of *NR4A1* upon SiO_2_ NP uptake.

In order to confirm the previous findings and to validate the RNA-seq results, the endocytosis related-genes clathrin light chain (*CLTC*), caveolin-1 (*CAV1*), early endosome antigen 1 (*EEA1*), lysosomal-associated membrane protein 1 (*LAMP1*), Rac family small GTPase 1 (*RAC1*), and dynamin 2 (*DNM2*) were evaluated at 1, 6, and 24 h after exposure to 60 and 200 nm SiO_2_ NPs by real-time qRT-PCR (Figure 4A). SiO_2_ NPs measuring 200 nm were included to investigate if particle size impacts cellular response at the gene level. No gene was found to be differentially expressed in A549 cells after exposure to either NPs. In MDM cells, *LAMP1* was down-regulated (Fold change = −1.7) upon 24 h exposure to 200 nm SiO_2_ NPs. As previously stated, lower co-localization values between 200 nm SiO_2_ NPs and lysosomes were observed after 24 h. In this regard, down-regulation of *LAMP1* might be associated with an impaired process of autophagic lysosome reformation [21,22,57]. Since *CAV1* is only weakly expressed in MDM cells, it was not included in the analysis. Nevertheless, and to confirm the results of RNA-Seq, the nuclear receptor subfamily 4 group A member 1 (*NR4A1*), one of the genes that was found to be up-regulated in MDM cells, was included in the real-time qRT-PCR analysis. The results confirmed the up-regulation of *NR4A1* with 60 nm SiO_2_ NPs after exposure for 6 h. In addition, we observed the up-regulation of this gene after 1 h, for 60 nm (Fold change = 2.3) and 200 nm SiO_2_ NPs (Fold change = 3.1), but not after 24 h, which suggests that *NR4A1* is an immediate-early response gene.

The comparison between protein and gene expression was evaluated by Western blot (Figure 4B). The results showed an increase of the expression for the NR4A1 protein in MDM cells at 6 h but not after 1 h. This can be explained by the fact that other regulation events occur between transcript and protein products [58]. The expression of the protein EEA1 in A549 cells was investigated and, as expected, its expression did not change considerably.

A computation model [25] was used to estimate the dose of SiO_2_ NPs that reaches the cells (i.e., delivered dose) (Figure S8). ISDD is a useful tool for calculating the delivered dose of NPs and performing more accurate analysis of cellular responses. A higher number of 60 nm SiO_2_ NPs reaches the cells in comparison with 200 nm SiO_2_ NPs after 1, 6, and 24 h. However, the obtained results do not directly correlate with the number of delivered NPs, as a higher *NR4A1* expression effect was observed after 1 h for the bigger NPs. It has been proved that the biologically most relevant dose metric for the evaluation of NP effects is the particle surface area [59]. When particle surface area was used as a metric, the simulation showed similar results, demonstrating a greater delivered dose for 60 nm SiO_2_ NPs. In brief, there is not a good correlation between cellular response and delivered dose in our study. This is due to the fact that the delivered dose is not the only factor influencing the cellular response. Other factors, such as the uptake mechanism and intracellular fate, can have an effect on biological responses [60].

### 3.3. Expression of NR4A1 in Macrophages upon Exposure to Au and PS NPs

**Figure 5:**
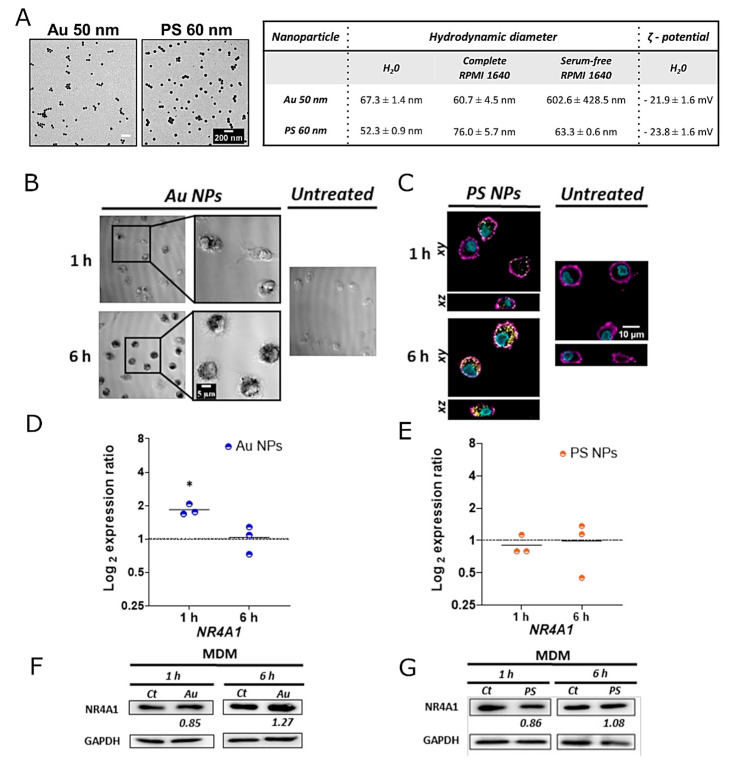
Effects of the cellular uptake of 50 nm Au and 60 nm PS in the expression of NR4A1 in primary human monocyte-derived macrophages (MDMs). (**A**) Representative transmission electron microscopy micrographs and physicochemical characterization of Au and PS NPs. Scale bar = 200 nm. Hydrodynamic diameter measured by dynamic light scattering in H_2_O, complete RPMI 1640 and serum-free RPMI 1640. (**B**) Differential interference contrast images showing the internalization of Au NPs in MDMs upon 1 and 6 h exposure. Scale bar = 5 µm. (**C**) Confocal laser scanning microscopy images revealing the uptake of PS NPs in MDM cells after exposure to PS NPs for 1 and 6 h. Scale bar = 10 µm. *NR4A1* gene expression upon exposure to Au NPs (**D**) and PS NPs. (**E**) NR4A1 protein expression after exposure to Au NPs (**F**) and PS NPs (**G**). In (**D**,**E**) comparisons between groups were performed with a paired *t*-test: * *p* ≤ 0.05. The representative Western blot images are shown in (**F**,**G**). The mean expression ratios of the indicated protein, determined via densitometry from three independent experiments, are shown at the bottom of each blot.

To evaluate the effect of NP material composition on the expression of NR4A1, MDMs were exposed to Au and PS NPs of similar size and shape for 1 and 6 h. Both NPs were characterized by TEM, DLS, and UV-Vis spectroscopy (Figure 5A and Figure S1). Similarly to SiO_2_ NPs, Au NPs of ca. 50 nm diameter are colloidally stable in complete RPMI 1640, but tend to aggregate in serum-free RPMI 1640. In contrast, the presence or absence of serum does not affect the stability of PS NPs of ca. 60 nm diameter. This can be explained by the fact that PS NPs possess a different surface chemistry than Au and SiO_2_ NPs, and the ionic strength of the cell-culture medium appears not to affect their stability.

Internalization of both NPs was confirmed at 1 and 6 h (Figure 5). Similarly to SiO_2_ NPs, *NR4A1* was up-regulated at early time points after exposure to Au NPs. The gene up-regulation was observed after 1 h but not after 6 h. A slight increase at the protein level was detected at 6 h, as also observed for SiO_2_ NPs. In contrast, exposure to PS NPs did not affect *NR4A1* expression. This can be explained by the fact that NPs possess different properties (e.g., material and surface chemistry), which per se can affect NP uptake. In addition, due to the different NP surface properties, distinct protein corona can be formed [57,61], which might also influence the internalization of NPs and, consequently, the signaling pathways. In vivo studies revealed that AuNPs tend to primarily accumulate in the liver and spleen [62]. Smaller NPs (< 8 nm) are cleared through renal clearance and larger ones via hepatobiliary excretion [48]. As shown, AuNPs can trigger inflammation, even at earlier stages, which requires a deep understanding of the cellular processes that are initiating this process. This knowledge is critical for the development of new NPs with optimized properties for optimal clearance and the ability to modulate and control inflammation.

## 4. Conclusions

In the present study, we confirm that the type of NP and the type of cell both influence NP uptake. Macrophages revealed a higher uptake rate for SiO_2_ NPs than with lung epithelial cells, which is attributed to the strong clearance, i.e., phagocytic, capability of macrophage cell types. Internalization of SiO_2_ NPs by lung epithelial cells was much slower and occurred to a lesser extent. Furthermore, cellular internalization of 60 nm SiO_2_ NPs did not lead to significant transcriptional changes after 6 h exposure to lung epithelial cells. In macrophages, despite our observation that genes related to endocytosis were not differentially expressed, we were able to identify the significant modification of the expression of gene *NR4A1*. The early up-regulation of *NR4A1* also occurred when macrophages were exposed for 1 h to 200 nm SiO_2_ and 50 nm Au NPs, which suggests that *NR4A1* is an immediate-early response gene for this type of NPs. NR4A1 is an important modulator of inflammatory response and it would be useful to investigate whether NR4A1 could be a potential therapeutic target to avoid exacerbated inflammation caused by NP uptake.

## Data Availability

The datasets underlying the results discussed in this article can be found in the online repository Zenodo under the doi 10.5281/zenodo.6125809 (Accessed on 18 February 2022).

## Supplementary Materials

The following supporting information can be downloaded at: www.mdpi.com/article/10.3390/nano12040690/s1, Figure S1: Characterization of the different NPs; Figure S2. Stability of SiO_2_ NPs in cell culture medium; Figure S3. Dye leaching study of SiO_2_-rhodamine B NPs; Figure S4. Phase-contrast images and cytotoxicity assay of lung epithelial cells (A549) and primary human monocyte-derived macrophages (MDMs) after exposure to SiO_2_ NPs; Figure S5. Cellular uptake of SiO_2_ NPs; Figure S6. Energy Dispersive X-ray (EDX) analysis of a cross-section of primary human monocyte-derived macrophages (MDMs) at three different spots (red cross), which confirms the intracellular presence of Silicon (Si); Figure S7. Gene set enrichment analysis (GSEA) with gene ontology (GO) biological processes (BP) of primary human monocyte-derived macrophages (MDMs) after 6 h exposure to 60 nm SiO_2_ NPs in comparison with untreated cells; Figure S8. Prediction of the effective cellular dose based on In vitro Sedimentation, Diffusion and Dosimetry (ISDD) model; Table S1. Information about the primers used for Real-time qRT-PCR.
