# Supplementary material for: Cellular Uptake of Silica and Gold Nanoparticles Induces Early Activation of Nuclear Receptor NR4A1
Source: Nanomaterials (Basel). 2022 Feb 18;12(4):690. doi: 10.3390/nano12040690 (PMC8877036; doi:10.3390/nano12040690)
Supplement: Supplementary file 1 [file nanomaterials-12-00690-s001.zip › nanomaterials-1552798-supplementary.pdf]

nanomaterials

MDPI

*Supplementary Materials*

# Cellular uptake of silica and gold nanoparticles induces early activation of nuclear receptor NR4A1

**Mauro Sousa de Almeida [1], Patricia Taladriz-Blanco [1,2], Barbara Drasler [1], Sandor Balog [1], Phattadon Yajan [1], Alke Petri-Fink [1,3] and Barbara Rothen-Rutishauser [1,*]**

[1] Adolphe Merkle Institute, University of Fribourg, Chemin des Verdiers 4, 1700 Fribourg, Switzerland; mauro.sousadealmeida@unifr.ch (M.S.d.A.); barbara.drasler@unifr.ch (B.D.); sandor.balog@unifr.ch (S.B.); phattadon.yajan@unifr.ch (P.Y.); alke.fink@unifr.ch (A.P.-F.); patricia.taladriz@inl.int (P.T.-B.);

[2] International Iberian Nanotechnology Laboratory (INL), Water Quality group, Av. Mestre José Veiga s/n, 4715-330 Braga, Portugal;

[3] Department of Chemistry, University of Fribourg, Chemin du Musée 9, 1700 Fribourg, Switzerland

* Correspondence: barbara.rothen@unifr.ch; Tel.: +41-26-300-9502

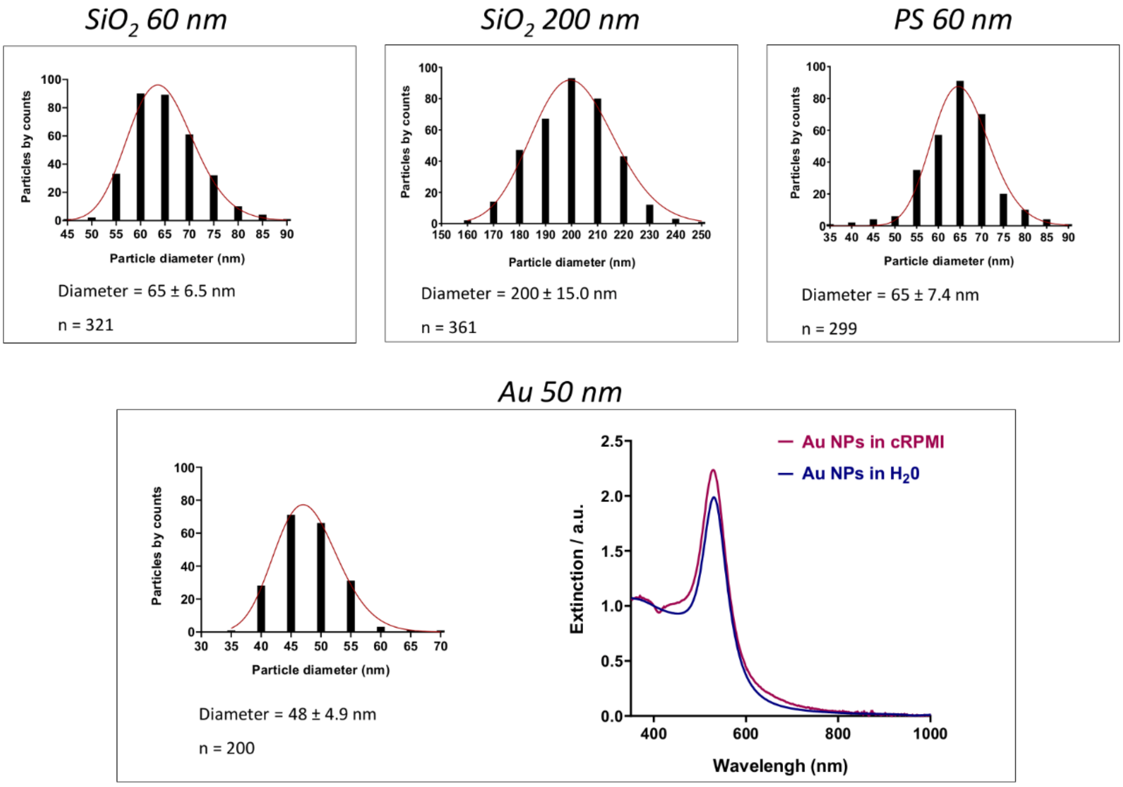


**Figure S1**. Characterization of the different NPs. Histograms showing the particle size distribution of various NPs determined by transmission electron microscope in H20. At the bottom right, UV-VIS spectra of Au NPs in H20 and complete RPMI 1640 (cRPMI).

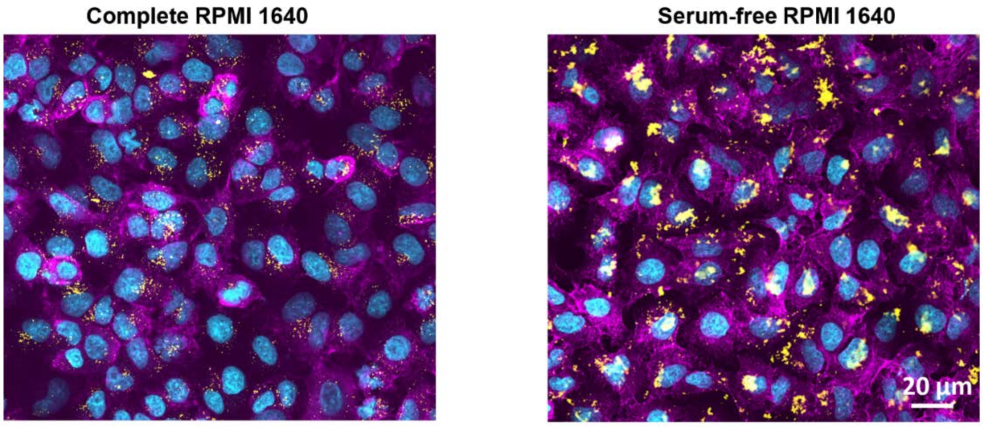


**Figure S2.** Stability of $SiO_2$ NPs in cell culture medium. Confocal laser scanning microscope micrographs showing the aggregation of 60 nm $SiO_2$ NPs (yellow) in serum-free RPMI 1640 (image on the right) after 4 h exposure to lung epithelial cells (A549). Cell nuclei (cyan) and cytoskeleton (magenta). Scale bar = 20 µm.

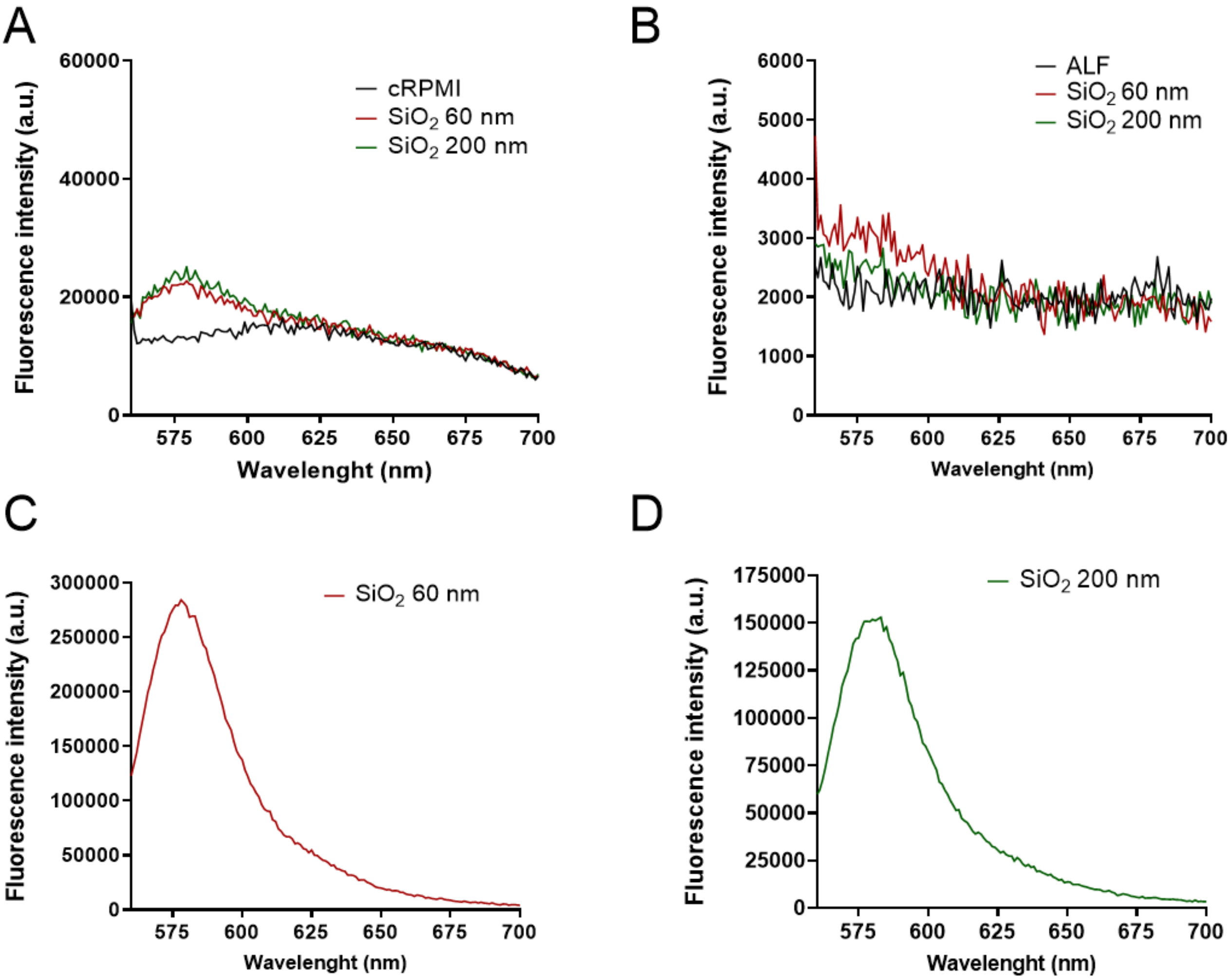


**Figure S3**. Dye leaching study of $SiO_2$-rhodamine B NPs. Fluorescence spectra of the supernatants after incubation of $SiO_2$ NPs in cRPMI (A) and artificial lysosomal fluid (ALF) (B) for 24h at 37°C (A). Supernatants reveal negligible fluorescence emission intensities. Fluorescence spectra of 60 nm (CB) and 200 nm (D) $SiO_2$ NPs in water, at 20 µg/mL.

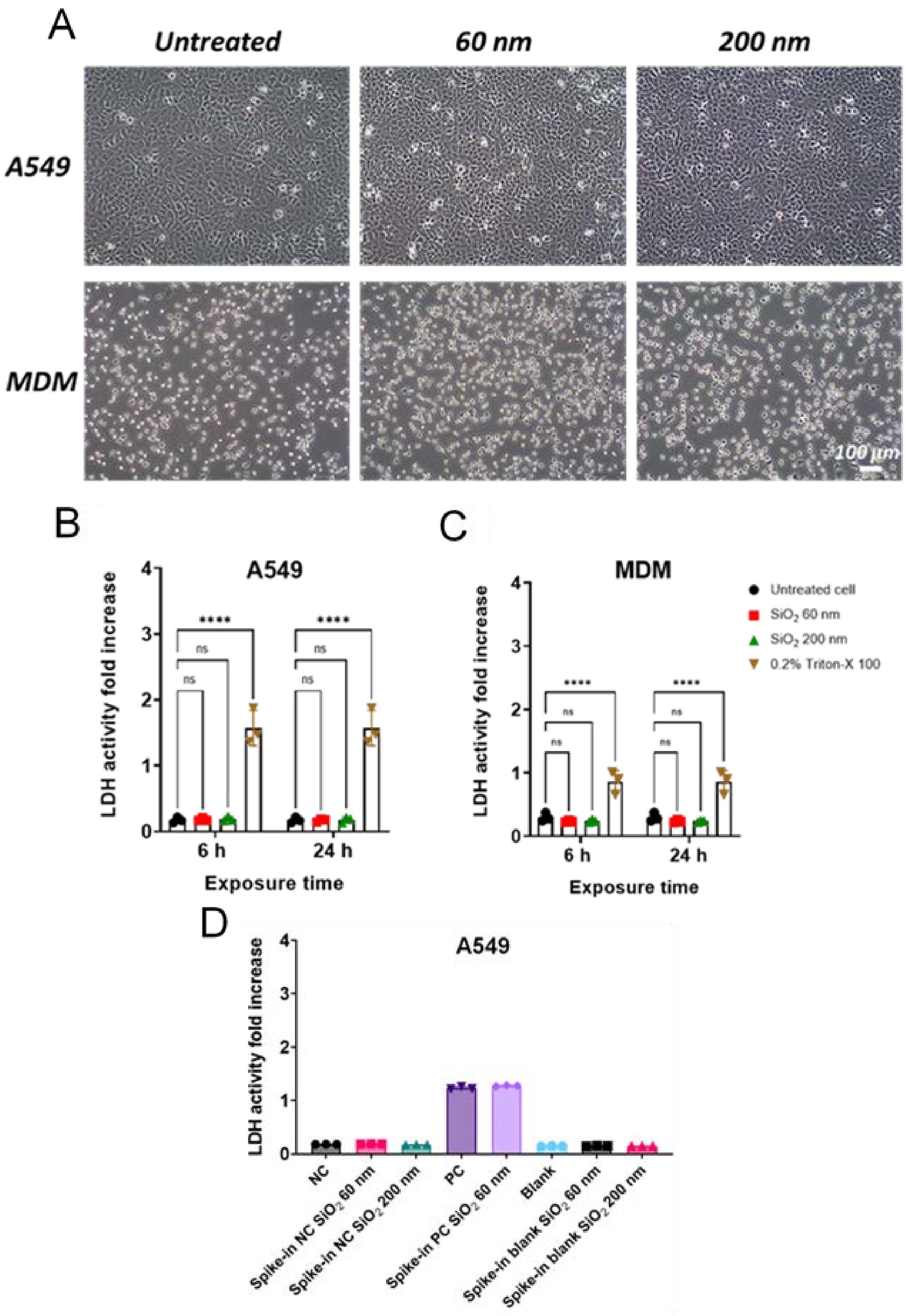


**Figure S4.** Phase-contrast images and cytotoxicity assay of lung epithelial cells (A549) and primary human monocyte-derived macrophages (MDMs) after exposure to $SiO_2$ NPs. Phase-contrast images of A549 and MDM cells after 24 h exposure to 60 and 200 nm $SiO_2$ NPs (A). Cytotoxicity assay (B-D) performed based on the lactate dehydrogenase (LDH) activity. No cytotoxic effect in A549 and (B) and MDMs (C) was observed for the tested NPs (20 µg/mL). Data from each experiment were obtained in triplicates and are presented as mean ± standard deviation. (Statistical significance determined by Dunnett's post-hoc test for multiple comparisons and represented by ****P ≤ 0.0001). ns: not significant. Interference analysis of $SiO_2$ NPs in LDH assay (D). Spike-in controls containing the same admistered dose (20µg/mL) were included and there was no evidence of quenching or auto-absorption. NC – Negative control; PC – Positive Control; Blank – cRPMI only. Data was obtained from one biological experiment with 3 technical replicates.

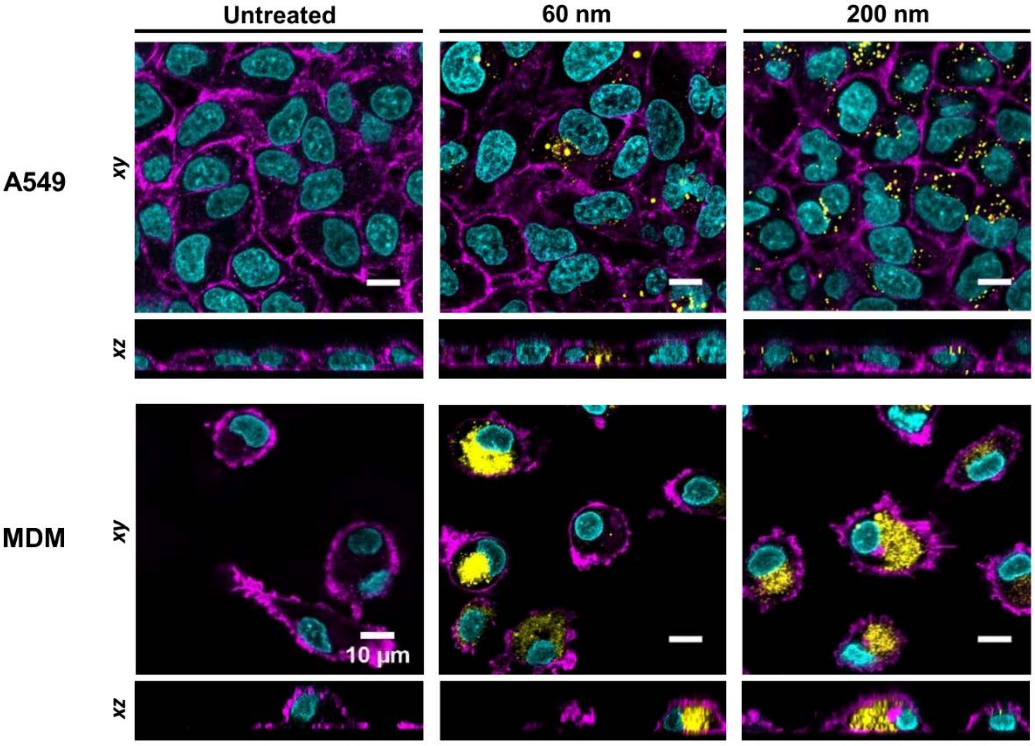


**Figure S5.** Cellular uptake of $SiO_2$ NPs. Confocal laser scanning microscope images revealing the internalization of 60 nm and 200 nm $SiO_2$ NPs upon 24 h exposure to lung epithelial cells (A549) and primary humam monocyte-derived macrophages (MDMs), at 20 µg/mL. Cell nuclei (cyan), cytoskeleton (magenta) and NPs (yellow). Scale bar = 10 µm.

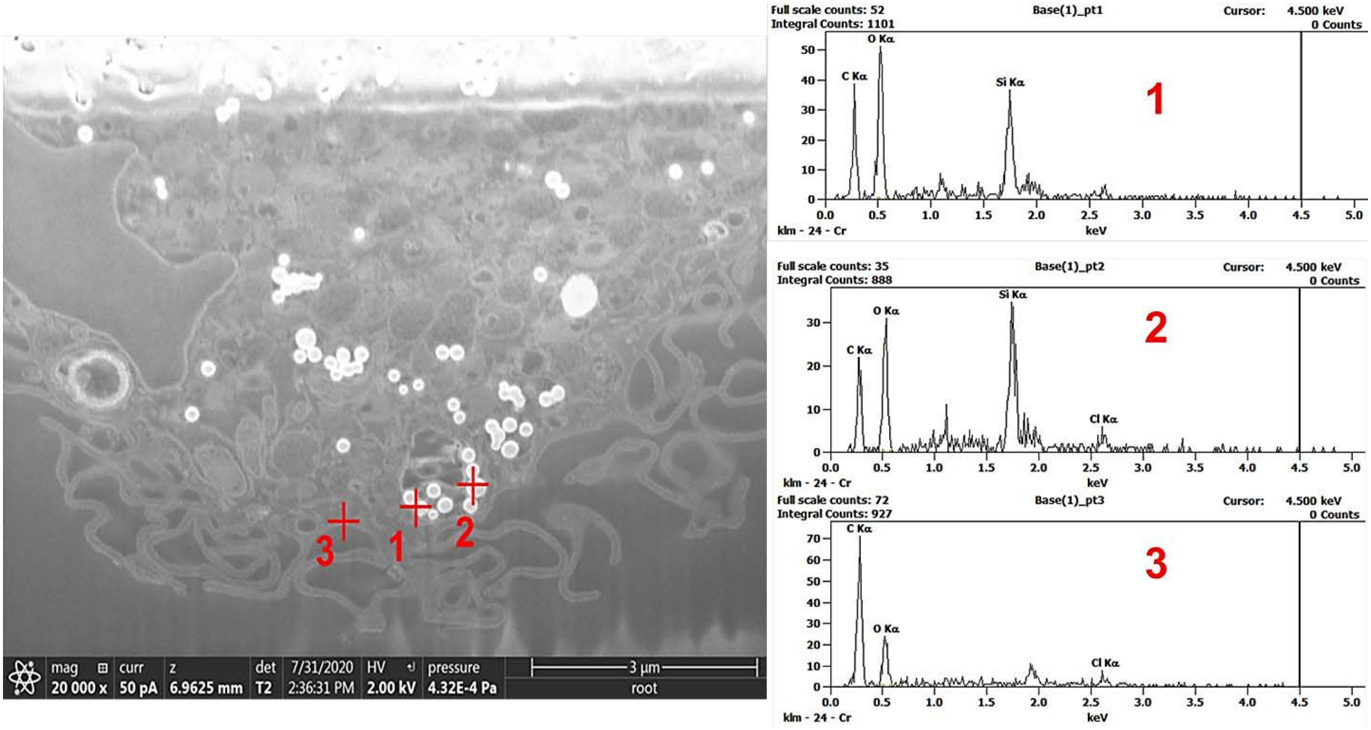


**Figure S6**. Energy Dispersive X-ray (EDX) analysis of a cross-section of macrophages (MDM) at three different spots (red cross), which confirms the intracellular presence of Silicon (Si).

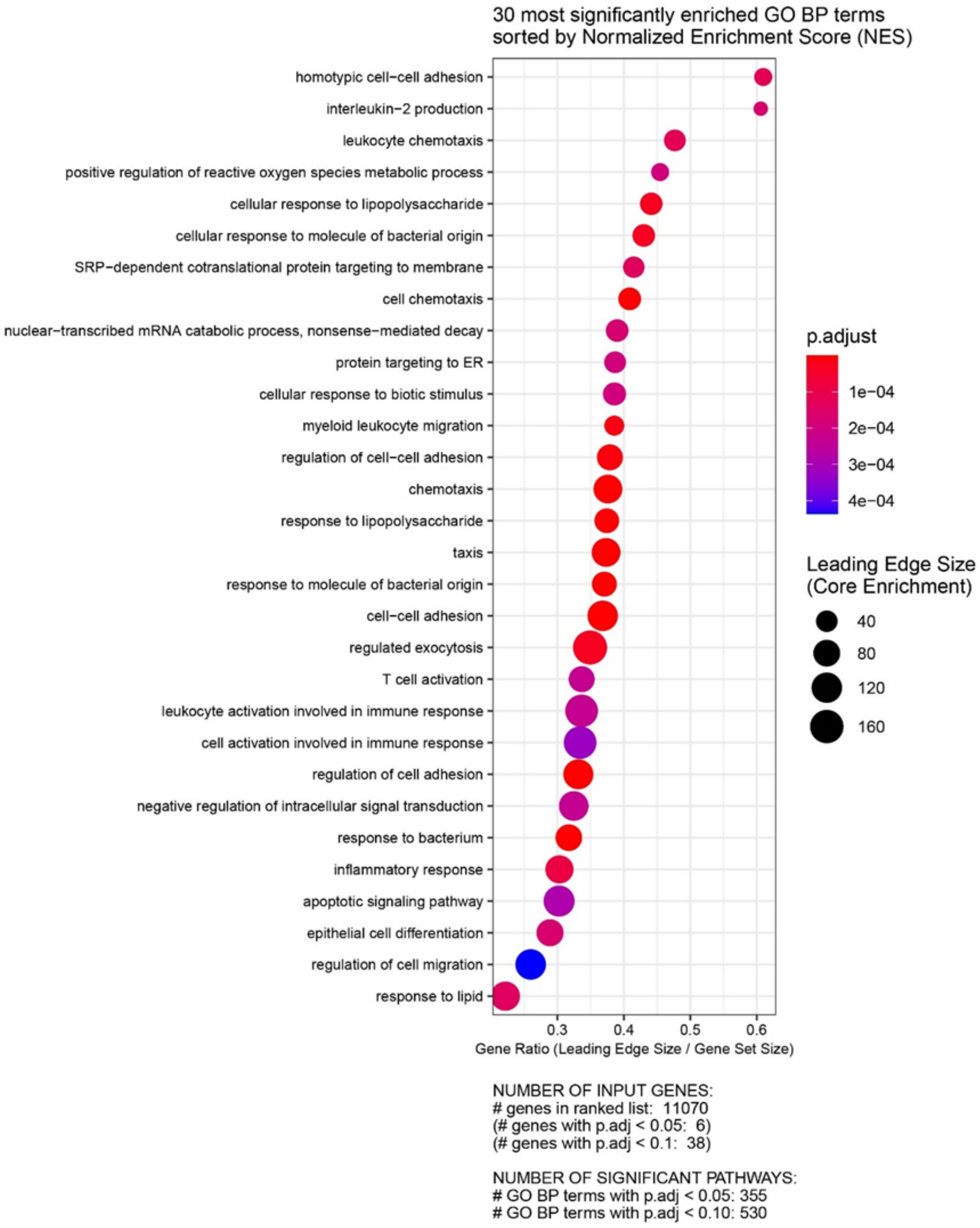


**Figure S7.** Gene set enrichment analysis (GSEA) with gene ontology (GO) biological processes (BP) of primary human monocyte-derived macrophages (MDMs) after 6 h exposure to 60 nm $SiO_2$ NPs in comparison with untreated cells.

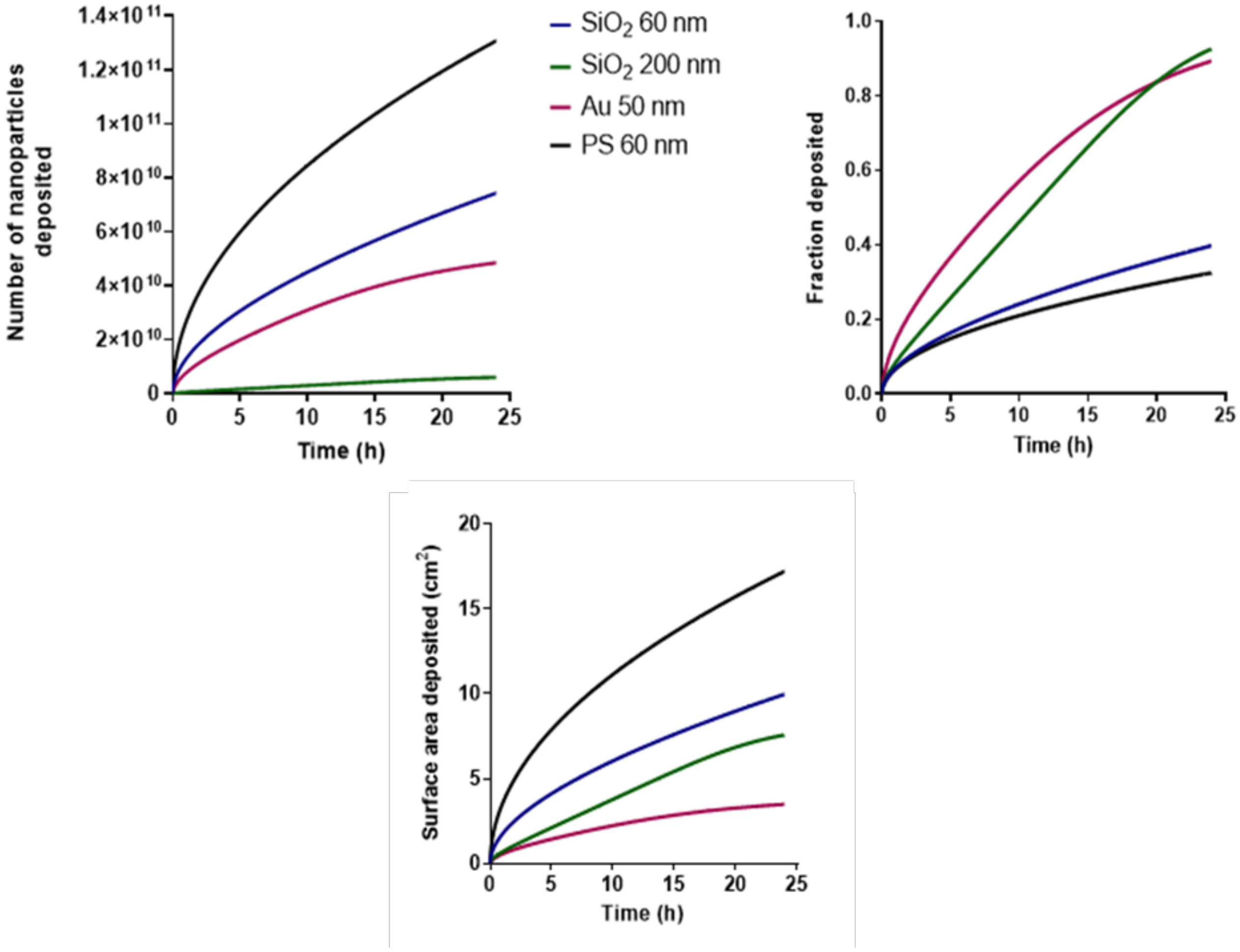


**Figure S8** - Prediction of the effective cellular dose based on In vitro Sedimentation, Diffusion and Dosimetry (ISDD) model. Number of nanoparticles deposited (top left), fraction deposited (top right) and surface area deposited (bottom) per $cm^2$.

**Table S1**. Information about the primers used for Real-time qRT-PCR. FW: Forward. RV: Reverse.

| *Primers* | *Gene symbol* | *Gene name* | *Sequence (5′->3′)* | *Product length* | *Efficiency* |
|---|---|---|---|---|---|
| ***Clathrin*** | *CLTC* | Clathrin heavy chain | FW: TTGCTGATGGTGCTGTCTCC<br>RV: CAGTGGTAGCTGTTTAACCTTGC | 78 | 1,85 |
| ***Dynamin*** | *DNM2* | Dynamin 2 | FW: GAGACAGAGCGAATCGTCACC<br>RV: TGTCGATCAGCAGAAGAATCTGG | 79 | 1,95 |
| ***Caveolin*** | *CAV1* | Caveolin 1 | FW: TGA GCG AGA AGC AAG TGT ACG<br>RV: GTGTGTCCCTTCTGGTTCTGC | 131 | 1,91 |
| ***Early endosome*** | *EEA1* | Early endosome antigen 1 | FW: ACATGACCTTGGAACGTGAGC<br>RV: GGCTTATTGTGGCCTCTGAGC | 96 | 1,92 |
| ***Lysosome*** | *LAMP1* | Lysosomal associated membrane protein 1 | FW: GCATAATGGCCAACTTCTCTGC<br>RV: CATCTGATGGCAGGTCAAAGG | 90 | 1,95 |
| ***Nuclear receptor*** | *NR4A1* | Nuclear receptor subfamily 4 group A member 1 | FW: GAAACCGCTGCCAGTTCTGC<br>RV: GCTGTCTGTTCGGACAACTTCC | 77 | 1,99 |

| | | | | | |
|---|---|---|---|---|---|
| ***Macropinocytosis / Phagocytosis*** | *RAC1* | Rac family small GTPase 1 | FW: TGGCTAAGGAGATTGGTGCTG<br>RV: CGGATCGCTTCGTCAAACAC | 88 | 1,93 |
| ***Housekeeping*** | *GAPDH* | Glyceraldehyde-3-phosphate dehydrogenase | FW: GTCGGAGTCAACGGATTTGG<br>RV: GCCATGGGTGGAATCATATTGG | 147 | 1,85 |
| | *YWHAZ* | Tyrosine 3-monooxygenase/tryptophan 5-monooxygenase activation protein zeta | FW: GCTGGTGATGACAAGAAAGGGAT<br>RV: GTTAAGGGCCAGACCCAGTC | 120 | 1,89 |
